# Phytochemical Profiling and Evaluation of Pharmacological Activities of *Hypericum scabrum* L.

**DOI:** 10.3390/molecules200611257

**Published:** 2015-06-18

**Authors:** Lan Jiang, Sodik Numonov, Khayrulla Bobakulov, Muhammad Nasimullah Qureshi, Haiqing Zhao, Haji Akber Aisa

**Affiliations:** 1 Key Laboratory of Plant Resources and Chemistry in Arid Regions, Xinjiang Technical Institute of Physics and Chemistry, Chinese Academy of Sciences, Urumqi 830011, China; E-Mails: jianglan@ms.xjb.ac.cn (L.J.); sodikjon82@gmail.com (S.N.); mnasimuq@yahoo.com (M.N.Q.); haiqing_zhq@126.com (H.Z.); 2 Key Laboratory of Xinjiang Indigenous Medicinal Plants Resource Utilization, Xinjiang Technical Institute of Physics and Chemistry, Chinese Academy of Sciences, Urumqi 830011, China; 3 University of Chinese Academy of Sciences, Beijing 100049, China; 4 State Scientifically-Experimental and Production Organization, Academy of Sciences of the Republic of Tajikistan, Dushanbe 734063, Tajikistan; 5 Institute of the Chemistry of Plant Substances, Academy of Sciences of the Republic of Uzbekistan, 100170 Tashkent, Uzbekistan; E-Mail: khayrulla@rambler.ru; 6 Department of Chemistry, Abdul Wali Khan University Mardan, Mardan 23200, Pakistan

**Keywords:** *Hypericum scabrum* L., ethylacetate fraction, column chromatography, NMR, ESI-MS, antidiabetic activity, antioxidant activity, antimicrobial activity

## Abstract

Phytochemical investigations of ethyl acetate-soluble part of the aerial part of *Hypericum scabrum* L. delivered eight pure phenolic compounds **1**–**8**. The pure compounds were identified through physico-chemical, NMR (1D, 2D) and mass spectrometric studies as: 3-8′′-bisapigenin (**1**), quercetin (**2**), quercetin-3-*O*-α-l-arabinofuranoside (**3**), quercetin-3-*O*-α-l-rhamnoside (**4**), quercetin-3-*O*-β-d-glucopyranoside (**5**), quercetin-3-*O*-β-d-galactopyranoside (**6**), (−)-epicatechin (**7**), (+)-catechin (**8**). Total polyphenolic compounds and total flavonoids contents were determined in the extract as 0.107 mg∙mg^−1^ and 0.023 mg∙mg^−1^ of the dried extract, respectively. Antioxidant activity using DPPH free radical scavenging assay delivered very strong activity for compounds **2** and **5**, **6** and crude extract **10**. Protein tyrosine phosphatase 1B (PTP-1B) inhibition experiment of isolated compounds and crude extracts resulted in significant inhibition activity for samples **2**, **7a**, **8a**, **11** and **12** with IC_50_ values ranging from 1.57 to 2.91 µM. Antimicrobial activity of the pure compounds and extracts produced average results against *Staphylococcus aureus*, *Escherichia coli* and *Candida albicans* strains. From our literature survey, it appears that all pure compounds except **2** were isolated and reported for the first time in *H. scabrum*.

## 1. Introduction

Medicinal plants have a long history of use in traditional systems of medicines, and are considered the primary sources of important medicines [1]. Flavonoids belong to a group of natural substances with variable phenolic structures and are found in fruit, vegetables, grains, bark, roots, stems, flowers, tea, and wine [2,3]. These natural products were known for their beneficial effects for health as crude plant material or plant extracts long before flavonoids were isolated in pure forms as effective compounds for various pharmacological activities such as antioxidant, antidiabetic, antimicrobial *etc.* [4,5]. Crude extracts of fruits, herbs, vegetables, cereals, nuts, and other plant materials rich in phenolic compounds are increasingly of interest in the food industry [6]. They are the group of compounds which received considerable attention from the researchers as depicted from the scientific literature. Mostly, they are present in plants as glycosides but can also be isolated in free aglycon form [7,8].

The genus Hypericum of the Hypericaceae consists of over 450 species, with worldwide distribution in warm temperate, subtropical and mountainous tropical regions [9], and a number of Hypericum species are widely used in folk and modern medicine. Hypericum species are medicinal plants known as healing herbs due to their various medicinal properties for the last two hundred years. Hypericin, an aromatic polycyclic anthrone, extracted from *Hypericum perforatum* L., has been shown to have potent, broad spectrum antimicrobial activity. This compound significantly inhibited the replication of several viruses, including HIV, influenza A, cytomegalovirus (CMV), Herpes simplex 1 and 2 (HSV-1 and HSV-2), and Epstein-Barr virus (EBV. *H. perforatum* L. oils have also shown notable biological activities including antiviral, wound healing, antioxidant, antimicrobial, antifungal, anxiolytic and anticonvulsant activities [9]. The essential oil compositions and antimicrobial activities of *Hypericum* have been recently reviewed [9]. Twenty-six components were characterized in the *H. scabrum* oil, accounting for 95.6% of the oil, which was dominated by α-pinene (44.8%). Other major components in *H. scabrum* oil were spathulenol (7.1%), verbenone (6.0%), *trans*-verbenol (3.9%), and γ-muurolene (3.5%). Thus, the *H. scabrum* oil from Tajikistan is qualitatively similar to essential oils from previous studies in that α-pinene was the major component, but does show some differences such as the absence of thymol, carvacrol, myrcene, or limonene [9]. The essential oil of the plants has been shown to possess anti-microbial and antioxidant activities and thus can be used in cosmetics, food and pharmaceutical industries [10]. *H. scabrum* aqueous extract showed remarkable anti-hypoxic and antidepressant effects thus, lend pharmacological justification to the use of the plant extract by traditional medicine practitioners.

*Hypericum scabrum* L. growing in Tajikistan had not been systemically investigated yet. Thus, the systematical study on its chemical constituents was undertaken since such an effort is an important route to discover bioactive compounds. The aim of this study was investigation of the chemical composition of the ethyl acetate fraction from the aboveground part of *Hypericum scabrum*. Further, the 2,2-diphenyl-1-picrylhydrazyl (DPPH) free radical scavenging activity, protein tyrosine phosphatase 1B (PTP-1B) inhibition assay and antimicrobial activity for pure compounds and also for crude extracts were determined. From our literature survey it appears that all the pure compounds except **2** were isolated and reported for the first time in *H. scabrum*.

## 2. Results and Discussion

### 2.1. Total Polyphenolic Compounds

Determination of total polyphenolic compounds were performed employing the Folin-Ciocalteau assay using gallic acid as the standard. Total polyphenolic compounds were calculated as equivalent of gallic acid. Seven point calibration curve was obtained after plotting the absorbance of the working standards against their respective concentrations using the Microsoft Excel sheet. The curve was passed through zero and this delivered a curve with *R*^2^ value as 0.9956. The amount of total polyphenolic compounds were calculated as 0.107 mg∙mg^−1^ of the dried extract using the regression equation obtained from the calibration curve.

### 2.2. Total Flavonoids contents

Aluminium-flavonoids complex formation assay was used for the quantification of contents of total flavonoids in the *Hypericum scabrum* extract. Seven points calibration curve was obtained with an *R*^2^ value as 0.9971 passing the curve through zero. Quantity of total flavonoids (x) was determined as 0.023 mg∙mg^−1^ of the dried extract using the straight line equation (y = 9.157x).

### 2.3. Spectral Identification of Isolated Compounds

**Figure 1:**
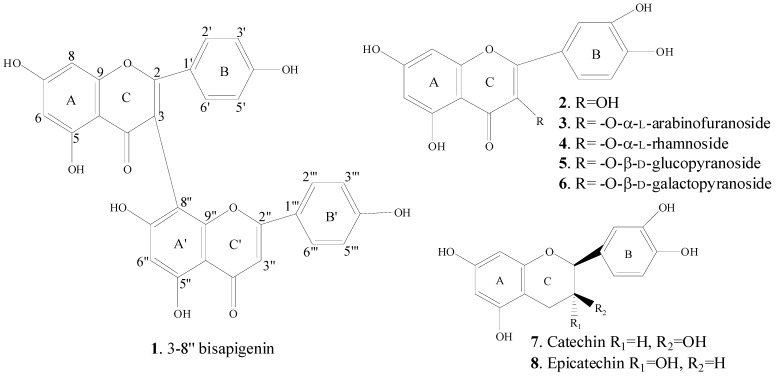
Structures of isolated compounds **1**–**8**.

The structures of compounds **1**–**8** were elucidated by MS and NMR spectral data analysis. Chemical structures of the isolated compounds are shown in Figure 1. The molecular ion observed at *m*/*z* 537.2 [M − H]^−^ recorded in negative ESI-MS mode together with ^1^H- and ^13^C-NMR data, including DEPT, suggesting the molecular formula C_30_H_18_O_10_ and consequently identified the compound **1** as 3-8′′-Bisapigenin (biflavonoid). At low field of the ^1^H-NMR spectra of **1** showed two broad singlet signals attributed to the six hydroxyl functionalities (δ 13.88 and 12.23 ppm). A pair of meta coupling proton signals observed at 6.75 (d, 1H, *J* = 1.7 Hz, H-6) and 6.81 (d, 1H, *J* = 1.7 Hz, H-8) for the A ring and another four doublets of symmetric proton signals at 8.01 (d, 2H, *J* = 8.4 Hz, H-2′, H-6′), 7.13 (d, 2H, *J* = 8.4 Hz, H-3′, H-5′), 7.94 (d, 2H, *J* = 8.4 Hz, H-2′′′, H-6′′′) and 7.16 (d, 2H, *J* = 8.4 Hz, H-3′′′, H-5′′′) showed the presence of an AA′BB′ coupling system, which indicated that 4′,4′′′-positions were substituted on the B and B′ rings, respectively. Two aromatic protons gave singlet signal at 6.85 ppm attributed to H-3′′ and H-6′′. The ^13^C-NMR data of **1** showed the presence of 30 carbon signals, which corresponded to 12 methines (δ_C_ 100.70 (C-6, C-6′′), 95.29 (C-8), 131.39 (C-2′, C-6′), 116.89 (C-3′, C-5′), 104.53 (C-3′′), 129.31 (C-2′′′, C-6′′′), 117.53 (C-3′′′, C-5′′′)), 18 quaternary carbons (δ_C_ 165.22 (C-2), 112.54 (C-3), 182.71 (C-4), 163.90 (C-5), 166.68 (C-7), 159.24 (C-9), 105.01 (C-10), 125.06 (C-1′), 162.20 (C-4′), 165.30 (C-2′′), 183.57 (C-4′′), 164.83 (C-5′′), 163.63 (C-7′′), 101.56 (C-8′′); 157.02 (C-9′′), 105.91 (C-10′′), 123.07 (C-1′′′), 163.23 (C-4′′′)). The linkage of two flavone units confirmed by analysis of the HMBC spectrum, which showed the cross-peak through four bond between H-6′′ (δ_H_ 6.85) and C-3 (δ_C_ 112.54). Furthermore, HMBC correlations were observed from H-2′, H-6′ to C-2, C-4′; H-6 to C-5, C-7, C-8, C-10; H-8 to C-8, C-7, C-9, C-10; H-3′′ to C-2′′, C-4′′, C-1′′′; H-6′′ to C-7′′, C-8′′, C-10′′. The above mentioned results revealed that the two flavone units were connected at C-3 and C-8′′ positions by C-C linkage in compound **1**.

The ^1^H-NMR spectrum of **2** (in Pyridine-*d_5_*; 400 MHz) showed three aromatic protons signals at 8.64 (1H, d, *J* = 2.2 Hz, H-2′), 7.41 (1H, d, *J* = 8.5 Hz, H-5′) and 8.14 (1H, dd, *J* = 8.5; 2.2 Hz, H-6′) in the form of an ABC spin-system suggesting a flavonol with 3′,4′-disubstituted B-ring and showed a pair of meta coupling proton signals at 6.74 (d, *J* = 2.0 Hz, H-6) and 6.78 (d, *J* = 2.0 Hz, H-8) for the A ring. The ^13^C-NMR spectra supported this hypothesis and showed 15 signals including carbonyl signal at (δ_C_ 177.77 (C-4). It revealed chemical shifts of carbon nucleus at δ_C_ 138.40 (C-3), 162.92 (C-5), 166.01 (C-7), 147.59 (C-3′), 150.14 (C-4′) that suggested the 3,5,7,3′,4′-oxygenated flavone nucleus. ESI-MS of compound **2** in negative ionization mode presented a molecular ion [M − H]^−^ at *m*/*z* value 301.0, suggesting the molecular formula C_15_H_10_O_7_ with the combination of ^1^H-NMR and ^13^C-NMR.

The ^1^H-NMR (Pyridine-*d*_5_; 400 MHz) of compound **3**, gave two meta-coupled doublets each with (*J* = 2.1), at 6.20 and 6.40, representing H-6 and H-8 of the A-ring. Three proton signals observed at 8.29 (1H, d, *J* = 2.2 Hz), 7.35 (1H, d, *J* = 8.5 Hz) and 7.99 (1H, dd, *J* = 8.4; 2.2 Hz), correspond to the H-2′, H-5′ and H-6′ protons of B-ring, respectively. The anomeric proton signal appeared as broad singlet at 6.52 ppm assigned to α-arabinose moiety. Two double of doublets corresponded to methylene protons at 4.10 (1H, dd, *J* = 11.9; 4.5 Hz, H-4a), 4.18 (1H, dd, *J* = 11.9; 3.7 Hz, H-4b), respectively. The connection of sugar moiety to quercetin was confirmed by HMBC correlation between H-1′′ and C-3. The negative ESI-MS of compound **3** gave the quasi-molecular ion at *m*/*z* 433.4 [M − H]^−^. Thus, its molecular formula was deduced to be C_20_H_18_O_11_ and structure of compound **3** was determined to be quercetin-3-*O*-α-l-arabinofuranoside.

In ^1^H-NMR (Pyridine-*d_5_*; 400 MHz) spectrum of compound **4**, three broad singlet signals and two doublets were observed in aromatic region at 8.04, 6.72, 6.67, 7.73 and 7.32 which were assigned to H-2′, H-6, H-8, H-6′ and H-5′, respectively. In addition, ^1^H-NMR spectrum of compound **4** shows singlet signal at δ 6.30 (1H, s, H-1′′) for anomeric proton of α-rhamnose moiety. In the high field of spectrum appeared the methyl proton signal as doublet at 1.50 (3H, d, *J* = 5.9 Hz, H-6′′). The ^13^C-NMR (Pyridine-*d_5_*; 100 MHz) spectrum revealed four carbon signals in the region of 72 to 74 ppm and one methyl carbon signal at 18.92 ppm typical for rhamnose unit. On the basis of HMBC correlation from H-1′′ to C-3, we established the connection of sugar moiety to aglicon at position C-3 of compound **4**. The structure was also confirmed by determination of ESI-MS (negative mode) giving [M − H]^−^ ion at *m/z* 447.5. Therefore, its molecular formula was deduced to be C_21_H_20_O_11_ with the combination of ^1^H-NMR and ^13^C-NMR.

The ^1^H-NMR spectrum of **5** exhibited signals from the H-6 and H-8 position of the flavone molecule at δ 6.20 (1H, d, *J* = 1.9 Hz, H-6) and 6.41 (1H, d, *J* = 1.9 Hz, H-8), respectively. One broad singlet at δ 7.58 (1H, br. s), two doublets (*J* = 8.6 Hz) at δ 6.85 and 7.59 ppm indicated the presence of H-2′, H-5′ and H-6′, respectively. The proton of hydroxyl group typical for 5-OH at δ 12.66 (1H, s) and seven broad singlet signals resonance at δ 10.88, 9.76, 9.25, 5.32, 5.10, 4.98, 4.30 ppm indicated the presence of hydroxyl groups in molecule. In addition, ^1^H-NMR signal at δ 5.48 was assigned to anomeric proton of sugar moiety. The sugar moiety was deduced to be attached at position C-3, on the basis of HMBC experiment cross-peak between H-1′′ (δ 5.48) and C-3 (δ 133.30). The anomeric configuration of glucose in **5** was concluded to be β-form from value of the coupling constant of H-1′′ (d, *J* = 7.5 Hz). In ^13^C-NMR spectrum of **5**, six carbon signals of glucose moiety were observed along with signals of quercetin. The molecular ion observed at *m/z* 463.5 in the ESI-MS negative mode. The above results support the structure of **5** as quercetin-3-*O*-β-d-glucopyranoside.

Compound **6** was obtained as a yellow amorphous powder, m. p. 245–246 °C. Comparison of the ^1^H- and ^13^C-NMR data for **5** and **6** indicated a close relationship for the two structures, although **5**, **6** were measured in DMSO-*d*_6_. The major difference between **5**, **6** were the value of R_f_ (0.48 for **5** and 0.45 mm for **6**), the proton multiplicity of sugar moiety and carbon chemical shift values of sugar moiety. Aglycon part of ^1^H-NMR spectrum of compound **6** displayed the typical signals for quercetin. The carbon signals were elucidated to be galactose unit. The position of the sugar moiety in quercetin was established from HMBC cross-peak between H-1′′ (δ 5.38) and C-3 (δ 133.45). Thus, the structure of compound **6** was determined to be quercetin-3-*O*-β-d-galactopyranoside.

The compounds **7**, **8** were obtained as a pale red powder. It gave one spot on TLC chromatogram (CHCl_3_/MeOH/H_2_O, 65:35:5). The compounds **7**, **8** on the basis ^1^H- and ^13^C-NMR were identified as a mixture of epicatechin (75%) and catechin (25%). The quantity of compounds **7** and **8** in sample were determined on the basis of integral intensities of the signals in ^1^H-NMR spectrum. Melting point of the mixture of standard epicatechin and catechin with the same ratio delivered the same result as of our purified compound.

### 2.4. Pharmacological Activities

Antidiabetic, antimicrobial and antioxidant activities of the six pure compounds (**1**–**6**) and seven crude extracts (**7a**, **8a**, **9a**, **10**–**13**) were determined. Pharmacological activities of the two pure compounds (**7**) and (**8**) could not be performed due to their low amounts which is not sufficient for these *in vitro* assays.

#### 2.4.1. PTP-1B Inhibition Screening

We have determined the effect of the pure isolated flavonoids (**1**–**6**) and the crude extract for their *in vitro* evaluation of percentage inhibition of the enzyme PTP-1B. Crude extracts **7a**, **8a**, **9a**, and **10**–**13** induced a PTP-1B enzymatic inhibition in a concentration-dependent manner with IC_50_ values 2.03 µM, 2.16 µM, 4.91 µM, 2.91 µM, 2.10 µM, 1.57 µM and 14.29 µM, respectively. The individual flavonoids except quercetin (IC_50_ value 2.19) have not shown good effect for PTP-1B inhibition. IC_50_ values of the all the samples are presented in Table 1. Seventy percent ethanol extract (sample **12**) showed highest antidiabetic activity (PTP-1B inhibition) with an IC_50_ 1.57 µM among the crude extracts and even more than the PTP-1B inhibitor. This significant activity in the initial extract with low activity in the fractionated extracts proved the synergistic relationship among the compounds in the 70% ethanol extract, which weakened after the separation of the synergistically bound analytes.

#### 2.4.2. *In Vitro* Antimicrobial Screening

The pure compounds **1**–**6** and crude extracts **7a**, **8a**, **9a**, **10**–**13** were evaluated through *in vitro* analysis for their antimicrobial activities and the results are tabulated in Table 1. Compounds were tested for antimicrobial activity against *Staphylococcus aureus* ATCC 6538 (Gram positive bacteria), *Escherichia coli* ATCC 11229 (Gram negative bacteria) and *Candida albicans* ATCC 10231 (Fungi) strains. All the pure compounds except the **3** and **4** were active against SA delivering highest zones of inhibition 12 for compound **2**. Zones of inhibition of compounds **1**, **5** and **6** were 5.5, 11 and 11, respectively, against SA. Compounds **3**, **4**, and **6** showed activity against EC with inhibition zones as: 5.5, 8 and 6.5, respectively. Among the six pure compounds, only compounds **2** and **4** delivered mild results for the inhibition of CA with zones of inhibition as 6.5 and 5.5, respectively. **8a**, **9a**, **11**–**13** did not show activity against any microbial strain while the crude extract **7a** showed activity with zones of inhibitions 7, 10 and 7 against SA, EC and CA, respectively. Sample **10** was only active against the EC and showed highest activity with zones of inhibitions as 12 among the crude extracts. The obtained results suggest that the tested compounds showed average and mild activity in comparison to the reference drugs Ampicillin and Amphotericin B.

#### 2.4.3. Antioxidant Activity

The free radical scavenging activity of the fractions was measured *in vitro* by 2,2-diphenyl-2-picrylhydrazyl (DPPH) assay. A lower IC_50_ value implied a better antioxidant activity of the tested samples. Quercetin (**2**) and quercetin glycoside (**5**) were the most active, with an IC_50_ value of 24.88 μM and 24.78 μM, respectively, which were even better than that of Vitamin C (IC_50_ 30.32 µM). Other glycosides also showed significant antiradical activities. The crude extracts **10** showed potent antiradical activities with IC_50_ value of 1.19 μg∙mL^−1^ and **8a**, **9a**, **11**–**13** showed relatively average antiradical activities with IC_50_ values 13.86, 24.62, 6.18, 10.81 and 16.29, respectively. The results of the antioxidant activity determined by the DPPH assay procedure using the standard Vitamin C, in pure compounds and crude extract with IC_50_ values are presented in Table 1. Results showed that ethyl acetate extract (sample **10**) delivered highest antioxidant activity with IC_50_ value of 1.19 μg∙mL^−1^ followed by *n*-butanol extract (sample **11**), which is completely in agreement with the previous literature [7,8].

**Table 1:** *In vitro* screening of IC_50_ values, inhibition zone (mm) PTP-1B inhibition and DPPH activities of the tested compounds **1**–**6** and **7a**, **8a**, **9a**, **10**–**13**.

Samples	Inhibition Zone Diameter (mm)			PTP-1B Inhibition; IC_50_ Values (µM)	DPPH Scavenging Effects; IC_50_ Values (µM)	DPPH Scavenging Effects; IC_50_ Values (μg∙mL^−1^)
	SA	EC	CA			
**1**	5.5	n.e.	n.e.	>50	>500	-
**2**	12	n.e.	6.5	2.19 ± 0.2	24.88 ± 1.4	-
**3**	n.e.	5.5	n.e.	>50	38.29 ± 2	-
**4**	n.e.	8	5.5	>50	32.10 ± 1.6	-
**5**	11	n.e.	n.e.	>50	24.78 ± 1.3	-
**6**	11	6.5	n.e.	>50	29.52 ± 1.5	-
**7a**	7	10	7	2.03 ± 0.1	-	>500
**8a**	n.e.	n.e.	n.e.	2.16 ± 0.1	-	13.86 ± 0.7
**9a**	n.e.	n.e.	n.e.	4.91 ± 0.3	-	24.62 ± 1.3
**10**	n.e.	12	n.e.	2.91 ± 0.2	-	1.19 ± 0.1
**11**	n.e.	n.e.	n.e.	2.1 ± 0.1	-	6.18 ± 0.3
**12**	n.e.	n.e.	n.e.	1.57 ± 0.1	-	10.81 ± 0.6
**13**	n.e.	n.e.	n.e.	14.29 ± 0.8	-	16.29 ± 0.8
Vitamin C	-	-	-	-	30.32 ± 2.4	5.34 ± 0.4
PTP-1B	-	-	-	1.97 ± 0.5	-	-
Ampicillin	19	14	-	-	-	-
Amphotericin B	-	-	15	-	-	-

IC_50_—half-maximal inhibitory concentration; SA—*Staphylococcus aureus* (ATCC 6538) bacteria strain; EC—*Escherichia coli* (ATCC 11229) bacteria strain; CA—*Candida albicans* (ATCC 10231) fungi strain; PTP-1B—enzyme inhibitor (3-(3,5-dibromo-4-hydroxy-benzoyl)-2-ethyl-benzofuran-6-sulfonicacid-(4-(thiazol-2-ylsulfamyl)-phenyl)-amide); n.e.—no effect; **7a**—petroleum ether & hexane extract (1:1); **8a**—hexane extract; **9a**—chloroform extract; **10**—ethyl acetate extract; **11**—*n*-butanol extract; **12**—70% ethanol extract; **13**—aqueous extract.

Our investigations clarified the use of crude extract as well as the flavonoids from *H. scabrum* for the development of formulations based on enzyme inhibition PTP-1B and antioxidants. Further confirmation of anti-diabetic and antioxidant activities of the extract and pure flavonoids from *H. scabrum* needs more research efforts, which may be applied in the food, agriculture and medicinal industry as a source of anti-diabetic and anti-oxidative agents. The underlying antimicrobial and antioxidant mechanisms of the flavonoids and crude extract, their contents in plant materials as well as their preparation on a large scale also need to be studied further.

## 3. Experimental Section

### 3.1. General Experimental Procedures

The NMR spectra were recorded on a Varian MR-400 (400 MHz for ^1^H and 100 MHz for ^13^C) and Varian VNMRS-600 (600 MHz for ^1^H and 150 MHz for ^13^C) spectrometers. Mass spectra were measured in a 2690-ZQ 4000 Water-Alliance LC-MS spectrometer (Applied Biosystems/MDS Sciex Concord, ON, Canada). Melting points were determined using a BUCHI Melting Point B-540 apparatus (Sigma-Aldrich, Darmstadt, Germany). Sephadex LH-20 gel (Amersham Pharmacia Biotech, Stockholm, Sweden) and Silica gel (100–300 mesh, Qingdao Haiyang Chemical Factory, Qingdao, China) were used for column chromatography. The fractions were monitored by TLC, and spots were visualized by heating Silica gel plates sprayed with 5% H_2_SO_4_ in EtOH.

### 3.2. Plant Material

Aerial parts of *H. scabrum* were collected from the Botanical Garden Academy of Sciences of the Republic of Tajikistan (38.5357500 N, 68.7790500 E and 767 m above sea level, Dushanbe, Tajikistan) during the flowering phase 15 June 2014. The plants were identified by Qurbonov Mansur. A voucher specimen has been deposited in Xinjiang Technical Institute of Physics and Chemistry, Urumqi, Chinese Academy of Sciences.

### 3.3. Determination of Total Polyphenolic Compounds and Total Contents of Flavonoids 

#### 3.3.1. Preparation of Sample

One gram of dried 70% ethanol extract of *Hypericum scabrum* was reconstituted in 20 mL of 70% methanol and this extract was used for the determination of total polyphenolic compounds and total flavonoids contents.

#### 3.3.2. Total Polyphenolic Compounds

Polyphenolic compounds were determined by Folin-Ciocalteau method [4] using gallic acid as the reference standard in the concentration range 0.02 mg∙mL^−1^ to 0.2 mg∙mL^−1^ in water. One milliliter standards/extract/blank (water) and 5 mL of diluted FC reagent (1:10 FC reagent to water) were mixed in a test tube. To each test tube 4 mL of Na_2_CO_3_ solution (7.5%) was added after 8 minutes and mixed. The test tubes were covered and allowed to react for 2 h at room temperature protecting them from strong light. Absorbances of these test solutions were determined at 740 nm against the prepared blank using UV-visible spectrophotometer.

#### 3.3.3. Determination of Total Flavonoids

Amount of total flavonoids were estimated using the procedure adopted by Chang *et al*. 2002 [11]. Quercetin was used standard and six working standard solutions were prepared in the concentration range: 0.01 mg mL^−1^ to 0.1 mg mL^−1^ in methanol, for constructing the calibration curve. Then, 0.5 mL of plant extract/standard solution/Blank (methanol) was mixed with 1.5 mL of methanol in a test tube. To the test tube, 0.1 mL of 10% aluminum chloride, 0.1 mL of 1 M potassium acetate and 2.8 mL of distilled water were added and mixed thoroughly after each addition. The solutions were stored for 30 min. at room temperature. The absorbances of the reaction mixtures were measured at 415 nm using the UV-visible spectrophotometer correcting the absorbance with the prepared blank solution.

### 3.4. Extraction, Isolation and Purification Procedures

The air-dried and powdered aerial part (2.65 kg) was extracted with a mixture of petroleum ether and hexane (1:1) several times to remove oil and fatty acids. The defatted plant material was then extracted with 70% ethanol (3 × 5 L) three times for 48 h at room temperature. The extract was concentrated under vacuum and dried to give the residue (475 g). The dried residue was suspended in water and successively partitioned in hexane, chloroform, ethyl acetate and *n*-butanol to afford the hexane fraction (45 g), CHCl_3_ fraction (58 g), EtOAc fraction (67 g), *n*-butanol fraction (45 g) and H_2_O fraction (105 g). Part of the ethyl acetate fraction (25 g) was subjected to silica gel column chromatography (100 to 200 mesh, 900 g), starting the elution with petroleum ether/EtOAc (1:1). The polarity was increased gradually to 100% EtOAc and ended with EtOAc/MeOH (4:1) affording 112 (1 to 112) fractions of 150 mL. The fractions were analyzed by silica gel thin layer chromatography (TLC) using the mobiles phases (CHCl_3_/MeOH/H_2_O, 65:35:5 and 73:24:4). Similar fractions were combined as: 13 to 48 (A), 50 to 58 (B), 59 to 68 (C), 69 to 80 (D) and 85 to 107 (E) delivering five main fractions.

**Figure 2:**
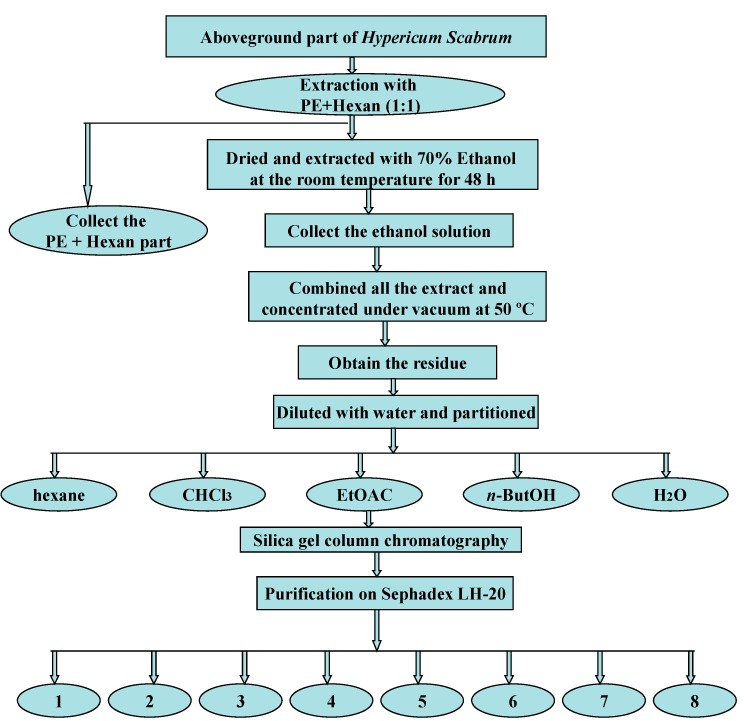
Flow sheet diagram of extraction, fractionation, isolation and purification of **1** to **8**.

Fraction A (6.0 g) was filtered on sephadex LH-20 using MeOH/Acetone (1:1) as mobile phase affording compound **1** (56 mg) and compound **2** (1.13 g). Fractions B and C (2.3; 1.6 g) were eluted on sephadex LH-20 using MeOH/H_2_O (9:1) and CH_2_CI_2_/MeOH (2:1) as mobile phase affording compound **3** (55 mg) and compound **4** (51 mg). From the fractions D (1.6 g) and E (800 mg) pure compounds **5** (39 mg) and **6** (1.4 g) were obtained through sephadex LH-20 column chromatography using eluting with MeOH/H_2_O in ratios of 4:1 and 1:1, respectively. The **7**, **8**, (11 mg) were isolated from the remainder fraction B with the eluent CHCI_3_: MeOH (7:1) through silica gel column chromatography (200 to 300 mesh). Figure 2 shows the flow sheet diagram of the extraction, fractionation, isolation and purification.

### 3.5. Mass Spectrometry

Mass spectrometric analyses of the pure compounds (**1** to **8**) were performed on a linear ion trap mass spectrometer (4000 Q TRAP) from AB Sciex. Mobile phase consisted of H_2_O and MeOH with a ratio of 20:80. MS was operated in negative ionization mode with ESI voltage −4500 V and the scanning was performed in the mass range *m*/*z* values from 100 to 1000. Two microliters of sample was injected and the capillary temperature was 450 °C.

### 3.6. Analytical Data of the Identified Compounds

*3-8′′-Bisapigenin* (**1**): yellow needle crystals (MeOH); M. p. 238–239 °C; ESI-MS, *m*/*z* 537.2 [M − H]^−^; ^1^H-NMR (400 MHz, Py-*d*_5_): δ 6.75 (d, 1H, *J* = 1.7 Hz, H-6), 6.81 (d, 1H, *J* = 1.7 Hz, H-8), 8.01 (d, 2H, *J* = 8.4 Hz, H-2′, H-6′), 7.13 (d, 2H, *J* = 8.4 Hz, H-3′, H-5′), 6.85 (s, 2H, H-3′′, H-6′′), 7.94 (d, 2H, *J* = 8.4 Hz, H-2′′′, H-6′′′), 7.16 (d, 2H, *J* = 8.4 Hz, H-3′′′, H-5′′′), 13.88 (br.s, 2H, OH-5, OH-5′′), 12.23 (br.s, 4H, OH); ^13^C-NMR (100 MHz, Py-*d*_5_): δ 165.22 (C-2), 112.54 (C-3), 182.71 (C-4), 163.90 (C-5), 100.70 (C-6), 166.68 (C-7), 95.29 (C-8), 159.24 (C-9), 105.01 (C-10), 125.06 (C-1′), 131.39 (C-2′, C-6′), 116.89 (C-3′, C-5′), 162.20 (C-4′), 165.30 (C-2′′), 104.53 (C-3′′), 183.57 (C-4′′), 164.83 (C-5′′), 100.70 (C-6′′), 163.63 (C-7′′), 101.56 (C-8′′), 157.02 (C-9′′), 105.91 (C-10′′), 123.07 (C-1′′′), 129.31 (C-2′′′, C-6′′′), 117.53 (C-3′′′, C-5′′′), 163.23 (C-4′′′). The ^1^H-NMR and ^13^C-NMR spectral data of compound **1** were consistent with the reported literature [12,13].

*Quercetin* (**2**): yellow needle crystals (MeOH); M. p. 315–316 °C; ESI-MS, *m*/*z* 301.0 [M − H]^−^; ^1^H-NMR (400 MHz, Py-*d*_5_): δ 6.74 (d, 1H, *J* = 2.0 Hz, H-6), 6.78 (d, 1H, *J* = 2.0 Hz, H-8), 7.41 (d, 1H, *J* = 8.5 Hz, H-5′), 8.14 (dd, 1H, *J* = 8.5; 2.2, Hz, H-6′), 8.64 (d, 1H, *J* = 2.2 Hz, H-2′), 13.36 (s, 1H, OH-5); ^13^C-NMR (100 MHz, Py-*d*_5_): δ 148.21 (C-2), 138.40 (C-3), 177.77 (C-4), 162.92 (C-5), 99.70 (C-6), 166.01 (C-7), 94.78 (C-8), 157.94 (C-9), 104.94 (C-10), 124.33 (C-1′), 117.1 (C-2′; C-5′), 147.59 (C-3′), 150.14 (C-4′), 121.53 (C-6′). The ^1^H-NMR and ^13^C-NMR spectral data of compound **2** were consistent with the reported literature [8].

*Quercetin-3-O-α-l-arabinofuranoside* (**3**): yellow needle crystals (MeOH/H_2_O); M.p. 231–232 °C; ESI-MS, *m*/*z* 433.4 [M − H]^−^; ^1^H-NMR (400 MHz, Py-*d*_5_): δ 6.72 (d, 1H, *J* = 2.1 Hz, H-6), 6.67 (d, 1H, *J* = 2.1 Hz, H-8), 7.35 (d, 1H, *J* = 8.4 Hz, H-5′), 7.99 (dd, 1H, *J* = 8.4; 2.2, Hz, H-6′), 8.29 (d, 1H, *J* = 2.2 Hz, H-2′), 13.31 (br.s, 1H, OH-5), 6.52 (br.s. 1H, H-1′′), 5.19 (d, 1H, *J* = 3.0 Hz, H-2′′), 4.90 (dd, 1H, *J* = 5.1; 3.1 Hz, H-3′′), 4.82 (m, 1H, H-4′′), 4.10 (dd, 1H, *J* = 11.9; 4.5 Hz, H-5′′), 4.18 (dd, 1H, *J* = 11.9; 3.7 Hz, H-5′′); ^13^C-NMR (100 MHz, Py-*d*_5_): δ 158.53 (C-2), 135.20 (C-3), 179.75 (C-4), 163.33(C-5), 100.24 (C-6), 166.41 (C-7), 95.08 (C-8), 158.17 (C-9), 105.72 (C-10), 122.90 (C-1′), 117.61 (C-2′), 147.61 (C-3′), 151.15 (C-4′), 117.14 (C-5′), 122.85 (C-6′), 110.29 (C-1′′), 83.94 (C-2′′), 79.44 (C-3′′), 89.13 (C-4′′), 62.96 (C-5′′) [identical with the literature] [14].

*Quercetin-3-O-α-l-rhamnoside* (**4**): yellow needle crystals (MeOH/H_2_O); M. p. 220–221 °C; ESI-MS, *m*/*z* 447.5 [M − H]^−^; ^1^H-NMR (400 MHz, Py-*d*_5_): δ 6.72 (s, 1H, H-6), 6.67(s, 1H, H-8), 7.32 (d, 1H, *J* = 8.3 Hz, H-5′), 7.73 (d, 1H, *J* = 8.3 Hz, H-6′), 8.04 (br.s. 1H, H-2′), 13.41 (br. s, 1H, OH-5), 6.30 (br. s. 1H, H-1′′), 4.67 (br. d, 1H, *J* = 9.2 Hz, H-2′′), 4.41 (m, 1H, H-3′′), 4.32 (t, 1H, *J* = 9.3 Hz, H-4′′), 5.10 (br. s. 1H, H-5′′), 1.50 (d, 3H, *J* = 5.9 Hz, H-6′′); ^13^C-NMR (100 MHz, Py-*d*_5_): δ 158.69 (C-2), 136.53 (C-3), 179.59 (C-4), 163.47 (C-5), 100.25 (C-6), 166.44(C-7), 95.06 (C-8), 158.20 (C-9), 105.92 (C-10), 122.80 (C-1′), 117.58 (C-2′), 147.84 (C-3′), 151.09 (C-4′), 117.00 (C-5′), 122.68 (C-6′), 104.54 (C-1′′), 73.07 (C-2′′), 72.61 (C-3′′), 73.87 (C-4′′), 72.53 (C-5′′), 18.92 (C-6′′) [identical with the literature] [14].

*Quercetin-3-O-β-d-glucopyranoside* (**5**): yellow needle crystals (MeOH/H_2_O); M. p. 231–232 °C; ESI-MS, *m*/*z* 463.5 [M − H]^−^; ^1^H-NMR (600 MHz, DMSO-*d*_6_): δ 6.20 (d, 1H, *J* = 1.9 Hz, H-6), 6.41(d, 1H, *J* = 1.9 Hz, H-8), 6.85 (d, 1H, *J* = 8.6 Hz, H-5′), 7.59 (d, 1H, *J* = 8.6 Hz, H-6′), 7.58 (br. s., 1H, H-2′), 12.66 (s, 1H, OH-5), 10.88 (br. s, 1H, OH-7), 9.76 (br. s, 1H, OH-3′), 9.25 (br. s, 1H, OH-4′), 5.32 (br. s, 1H, OH-3′′), 5.10 (br. s, 1H, OH-4′′), 4.98 (br. s, 1H, OH-4′′), 4.30 (br. s, 1H, OH-6′′), 5.48 (d, 1H, *J* = 7.5 Hz, H-1′′), 3.23 (m, 2H, H-2′′, H-3′′), 3.08 (m, 2H, H-4′′, H-5′′), 3.32 (d, 1H, *J* = 11.5 Hz, H-6′′), 3.58 (dd, 1H, *J* = 11.5; 3.1 Hz, H-6′′); ^13^C-NMR (150 MHz, DMSO-*d*_6_): δ 156.17 (C-2), 133.30 (C-3), 177.45 (C-4), 161.26 (C-5), 98.67 (C-6), 164.13(C-7), 93.51(C-8), 156.33 (C-9), 103.99 (C-10), 121.17 (C-1′), 116.20 (C-2′), 144.83 (C-3′), 148.47 (C-4′), 115.21 (C-5′), 121.62 (C-6′), 100.82 (C-1′′), 74.10 (C-2′′), 76.51 (C-3′′), 69.94 (C-4′′), 77.63 (C-5′′), 60.98 (C-6′′) [identical with the literature] [15,16].

*Quercetin-3-O-β-d-galactopyranoside* (**6**): yellow powder (MeOH/H_2_O); M. p. 245–246 °C; ESI-MS, *m*/*z* 463.5 [M − H]^−^; ^1^H-NMR (600 MHz, DMSO-*d*_6_): δ 6.20 (d, 1H, *J* = 2.0 Hz, H-6), 6.40(d, 1H, *J* = 2.0 Hz, H-8), 6.81 (d, 1H, *J* = 8.5 Hz, H-5′), 7.67 (dd, 1H, *J* = 8.5; 2.2 Hz H-6′), 7.52 (d, 1H, *J* = 2.2 Hz H-2′), 12.64 (s, 1H, OH-5), 10.89 (br.s, 1H, OH-7), 9.76 (br.s, 1H, OH-3′), 9.18 (br.s, 1H, OH-4′), 5.15 (br. s, 1H, OH), 4.88 (br. s, 1H, OH), 4.45 (br. s, 2H, OH), 5.38 (d, 1H, *J* = 7.7 Hz, H-1′′), 3.57 (dd, 1H, *J* = 9.4; 7.2 Hz, H-2′′), 3.37 (dd, 1H, *J* = 9.4; 3.3 H-3′′), 3.65 (d, 1H, *J* = 3.3 Hz, H-4′′), 3.33 (t, 1H, *J* = 6.0 H-5′′), 3.29 (dd, 1H, *J* = 10.4; 6.0 Hz, H-6′′), 3.45 (dd, 1H, *J* = 10.4; 6.0 Hz, H-6′′); ^13^C-NMR (150 MHz, DMSO-*d*_6_): δ 156.20 (C-2), 133.45 (C-3), 177.46 (C-4), 161.22 (C-5), 98.68 (C-6), 164.20 (C-7), 93.50 (C-8), 156.29 (C-9), 103.88 (C-10), 121.08 (C-1′), 115.90 (C-2′), 144.83 (C-3′), 148.46 (C-4′), 115.17 (C-5′), 122.01 (C-6′), 101.76 (C-1′′), 71.19 (C-2′′), 73.17 (C-3′′), 67.91 (C-4′′), 75.85 (C-5′′), 60.13 (C-6′′). The ^1^H-NMR and ^13^C-NMR spectral data of compound **6** were consistent with the reported literature [16].

*(−)-Epicatechin* (**7**): pale red powder (MeOH); M. p. 228–229 °C; ESI-MS, *m*/*z* 289.0 [M − H]^−^; ^1^H-NMR (400 MHz, Py-*d*_5_): δ 5.40 (br. s., 1H, H-2), 4.75 (br. s., 1H, H-3), 3.44 (dd, 1H, *J* = 16.4; 4.6 Hz, H-4a), 3.57 (dd, 1H, *J* = 16.4; 3.7 Hz, H-4b), 6.71 (d, 1H, *J* = 2.3 Hz, H-6), 6.69(d, 1H, *J* = 2.3 Hz, H-8), 7.30 (d, 1H, *J* = 8.1 Hz, H-5′), 7.37 (dd, 1H, *J* = 8.1; 2.0 Hz, H-6′), 7.95 (d, 1H, *J* = 2.0 Hz, H-2′), 11.26 (br. s., 4H, OH), 11.47 (br. s., 1H, OH); ^13^C-NMR (100 MHz, Py-*d*_5_): δ 80.59 (C-2), 67.43 (C-3), 30.18 (C-4), 159.20 (C-5), 97.14 (C-6), 159.12 (C-7), 96.28 (C-8), 158.12 (C-9), 100.68 (C-10), 132.64 (C-1′), 116.85 (C-2′), 147.43 (C-3′), 147.33 (C-4′), 116.60 (C-5′), 119.89 (C-6′). The ^1^H-NMR and ^13^C-NMR spectral data of compound **7** were consistent with the reported literature [17,18,19].

*(+)-Catechin* (**8**): pale red powder (MeOH); M. p. 228–229 °C; ESI-MS, *m*/*z* 289.0 [M − H]^−^; ^1^H-NMR (400 MHz, Py-*d*_5_): δ 5.26 (d, 1H, *J* = 7.7 Hz, H-2), 4.66 (m, 1H, H-3), 3.37 (dd, 1H, *J* = 16.0; 8.4 Hz, H-4a), 3.73 (dd, 1H, *J* = 16.0; 5.5 Hz, H-4b), 6.73 (d, 1H, *J* = 2.3 Hz, H-6), 6.68(d, 1H, *J* = 2.3 Hz, H-8), 7.26 (m, 2H, H-5′, H-6′), 7.68 (d, 1H, *J* = 1.6 Hz, H-2′), 11.56 (br. s., 1H, OH); ^13^C-NMR (100 MHz, Py-*d*_5_): δ 83.79 (C-2), 68.71 (C-3), 30.24 (C-4), 158.89 (C-5), 97.14 (C-6), 159.27 (C-7), 96.02 (C-8), 157.85 (C-9), 101.53 (C-10), 132.67 (C-1′), 116.85 (C-2′), 147.69 (C-3′), 147.67 (C-4′), 116.65 (C-5′), 120.22 (C-6′). The ^1^H-NMR and ^13^C-NMR spectral data of compound **8** were consistent with the reported literature [17,18,19].

### 3.7. Protein Tyrosine Phosphatase 1B (PTP-1B) Inhibition Assay

The individual compounds **1** to **6** and crude extracts **7a**, **8a**, **9a**, **10** to **13** were tested for the measurement of PTP-1B activity using pNPP (p-nitrophenyl phosphate disodium salt) as substrate. Compounds were pre-incubated with the enzyme at room temperature for 5 min. Then, 178 µL of buffer solution (20 mM HEPES, 150 mM NaCl, 1 mM EDTA) was added in 96-well plates. One microliter of PTP-1B protein solution (0.115 mg∙mL^−1^) was added to the buffer solution. After that, 1 µL of test and positive control sample were added. Thereafter, 20 µL of the substrate pNPP (35 mM) was added and mixed for 10 min. The plate was incubated in the dark for 30 min and the reaction was terminated by adding 10 µL of 3 M NaOH. The absorbance was then determined at 405 nm wavelength. The system does not contain the enzyme solution in a blank, using a micro plate reader Spectra Max MD5 (USA Molecular Devices) absorption was measured at 405 nm. Inhibition (%) = [(OD_405_ − OD_405_ blank)/OD_405_ blank] × 100. IC_50_ was calculated from the percentage inhibition values.

### 3.8. Antimicrobial Activity

Antimicrobial activity of compounds **1** to **6** and crude extracts **7a**, **8a**, **9a**; **10** to **13** were measured using the agar well diffusion method. Bacterial and fungal pathogens such as *S. aureus* (ATCC 6538), *E. coli* (ATCC 11229) and *C. albicans* (ATCC 10231) were used as indicator strains for this analysis. These microorganisms were aseptically inoculated into appropriate liquid media and incubated at 37 °C. After 16 h, the cells were centrifuged at 6000 rpm for 10 min and then suspended in sterile water. The different cells (1 mL) were added to appropriate agar media (100 mL) prior to plating, and the wells were made using an agar well borer. To these wells, different concentrations of compounds **1** to **6** and the extracts **7a**, **8a**, **9a**; **10** to **13** were added and subsequently incubated at 37 °C for 24 h. Zones of inhibition were estimated by measuring the diameter of the microbial growth inhibition zones. Values were averaged from three independent experiments.

### 3.9. DPPH Radical Scavenging Assay

Radical scavenging assay was determined by a micro plate spectrophotometric method based on the reduction of DPPH according to the previous report [20]. Various dilutions of pure compounds and crude extracts in the concentration range 0.0625 to 1 mM and 0.0625 to 1 mg∙mL^−1^, respectively were made. Briefly DPPH solution (100 μL) and pure compound or crude extract in DMSO (100 μL) were added to each well of the micro plate and mixed. The mixture was shaken vigorously and left to stand at 37 °C for 30 min in dark. The absorbance of the solution was then measured at wavelength 515 nm using a micro plate spectrophotometer. Inhibition (%) of free radical (DPPH) was determined as [(A_control_ − A_sample_)/A_control_] × 100, where: A_control_ is the absorbance of the control sample containing all reagents except the test sample and A_sample_ is the absorbance of the test sample (pure compound or crude extract). Vitamin C was used as the positive control and tests were carried out in triplicate. The IC_50_ values were determined for all tested flavonoids, extract and control standard antioxidant. The IC_50_ value was defined as the concentration (in μM and μg∙mL^−1^) of pure compound and extracts that inhibits the formation of radicals by 50%.

## 4. Conclusions

The study confirmed the presence of bioactive compounds like quercetin glycosides, bisapigenin, catechin and epicatechin in the aerial part of *H. scabrum* in sufficient amounts to be isolated employing routinely applied chromatographic procedures. Seventy percent ethanol extract delivered best results for PTP-1B assay with IC_50_ 1.57 µM among the crude extracts while quercetin showed significant inhibition activity (IC_50_ 2.19 µM) for this enzyme. Quercetin (**2**) and quercetin glycoside (**5**) yielded strong DPPH radical scavenging activity among the isolated compounds. Our study suggests the use of aerial part of *H. scabrum* for antioxidant and antidiabetic formulations based on their proven activities. From our literature survey, it appears that all the pure compounds except **2** were isolated and reported for the first time in *H. scabrum*.
